# Microarray expression profile analysis of mRNAs and long non-coding RNAs in pulmonary tuberculosis with different traditional Chinese medicine syndromes

**DOI:** 10.1186/s12906-016-1436-y

**Published:** 2016-11-17

**Authors:** Ting-Ting Jiang, Li-Liang Wei, Li-Ying Shi, Zhong-Liang Chen, Chong Wang, Chang-Ming Liu, Zhong-Jie Li, Ji-Cheng Li

**Affiliations:** 1 Institute of Cell Biology, Zhejiang University, Hangzhou, Zhejiang China; 2 Department of Pneumology, Shaoxing Municipal Hospital, Shaoxing, Zhejiang China; 3 Department of Clinical Laboratory, Zhejiang Hospital, Hangzhou, Zhejiang China

**Keywords:** Pulmonary tuberculosis, TCM syndrome, mRNAs, lncRNAs

## Abstract

**Background:**

Combination chemotherapy with Western anti-tuberculosis (TB) drugs is the mainstay of TB treatment. Chinese herbal medicines with either heat clearing and detoxifying effects or nourishing Yin and reducing fire effects have been used to treat TB based on the Traditional Chinese Medicine (TCM) syndromes of TB patients. This study analyzed the expression profiles of long non-coding RNAs (lncRNAs) and mRNAs in TB patients with different TCM syndromes.

**Methods:**

TB patients were classified as pulmonary Yin deficiency (PYD) syndrome, hyperactivity of fire due to Yin deficiency (HFYD) syndrome, and deficiency of Qi and Yin (DQY) syndrome. Total RNA from 44 TB patients and healthy controls was extracted and hybridized with a human lncRNA microarray containing 30586 lncRNAs and 26109 mRNAs probes. Bioinformatics analyses, including gene ontology (GO) and pathways, were performed. Related clinical data were also analyzed.

**Results:**

Differentially expressed mRNAs and lncRNAs were identified (fold change >2, and *P* < 0.05) in PYD (634 mRNAs and 566 lncRNAs), HFYD (47 mRNAs and 55 lncRNAs), and DQY (63 mRNAs and 60 lncRNAs) patients. The most enriched pathways were the hippo signaling pathway (*P* = 0.000164) and the protein digestion and absorption pathway (*P* = 5.89017E-05). Clinical analyses revealed that the lipid indexes of TB patients were abnormal and that the triglyceride concentration was significantly higher in DQY patients (*P* = 0.0252). Our study is the first to acquire the microarray expression profiles of lncRNAs and mRNAs and analyze pathway enrichment in PYD, HFYD, and DQY patients with TB.

**Conclusions:**

Our analyses of the expression profiles of lncRNAs and mRNAs may represent a novel method to explore the biological essence of TCM syndromes of TB.

**Electronic supplementary material:**

The online version of this article (doi:10.1186/s12906-016-1436-y) contains supplementary material, which is available to authorized users.

## Background

Pulmonary tuberculosis (TB) caused by *Mycobacterium tuberculosis* (Mtb) infection is a leading cause of death. Nine million new TB patients and 1.5 million TB deaths occurred globally in 2013 [1]. TB remains a public threat to human health in China. Combination chemotherapy with anti-TB drugs (isoniazid, rifampicin, pyrazinamide and ethambutol for 2 months and isoniazid and rifampicin for 4 months) is the mainstay of TB treatment [2]. Most TB cases are cured using routine anti-TB therapy, but some TB patients may develop severe side effects [3–5] or drug-resistant TB [4, 6]. The adverse effects of anti-TB drugs vary greatly among individuals [2, 7], and these effects are closely related to disease progression and the immune status of the patient [8]. Individualized treatments that strengthen the body’s immune system and enhance the efficacy and reduce the toxicity of anti-TB drugs are a new method of treating TB [2, 8].

With more than 3000 years of clinical practice, Traditional Chinese Medicine (TCM) is a fully institutionalized medical system in China [9] and has been used to treat TB for at least 500 years [10]. TCM enables individualized health care [11, 12]. Diagnoses are based on the integrity of the body and TCM syndrome differentiation, and different patients receive different prescriptions [11, 12]. The TCM syndrome is the temporary state of the patient’s comprehensive response and is the premise for treatment [13]. Disease progression and the extent of damage are generally assessed by inspection, auscultation, olfaction, interrogation, and palpation in TCM [14, 15]. Patients with the same disease can undergo different TCM syndromes, thus providing an opportunity for personalized medicine [14–16].

TB patients have been classified into three main TCM syndromes: pulmonary Yin deficiency syndrome (PYD), hyperactivity of fire due to Yin deficiency syndrome (HFYD), and deficiency of Qi and Yin syndrome (DQY) [17]. Modern medical studies have shown that the integration of Chinese and Western medicine based on the TCM syndromes of TB patients can enhance the efficacy and reduce the side effects of anti-TB drugs and improve the immune response [18–20]. For example, Chinese herbs with heat-clearing and detoxifying effects or nourishing Yin and lowering fire effects, such as *Astragalus membranaceus* and *Radix Paeoniae Rubra (Chishao)*, have been used to treat TB [19–21]. Extracts from *Astragalus membranaceus* greatly improve the phagocytosis of mycobacteria [19, 21]. Extracts from *Radix Paeoniae Rubra* elevate the level of interleukin-8 [20] and drive the recruitment of T lymphocytes and neutrophils at infection sites to increase the bacteriostatic function of neutrophils [22, 23]. Extracts from *Prunella vulgaris* L. and *Radix Sophorae Flavescentis* have been shown to strengthen cell-mediated immunity in a rat model of multidrug-resistant TB [24].

However, TCM syndrome classification depends heavily on the clinical experience of TCM practitioners, and relevant fundamental experimental studies are lacking [15, 25]. The current study used Arraystar Human LncRNA Microarray technology to investigate the differential expression profiles of mRNAs and lncRNAs in PYD, HFYD, and DQY patients with pulmonary TB. The pathway enrichment of differentially expressed mRNAs and clinical indexes were also analyzed using bioinformatics methods.

## Methods

### Patients and control subjects

A total of 292 pretreated TB patients (aged 18 to 75 years), including 92 PYD cases, 124 HFYD cases, and 76 DQY cases, from Shaoxing Municipal Hospital (Shaoxing, Zhejiang, China) were included in the current study. All recruited TB patients were diagnosed according to the diagnostic criteria of the Ministry of Health, China, and met one of the following diagnostic criteria: positive sputum culture or smear; typical active TB findings on chest X-ray and CT scan; or pulmonary pathological lesions diagnosed as TB. TB cases with other diseases, such as hepatitis B, diabetes, extra-pulmonary TB, AIDS, and immune inhibitor users, were excluded. TB patients were classified into PYD, HFYD, DQY syndromes according to the ‘Standard of disease diagnosis and curative effect of Traditional Chinese Medicine’ [18]. A total of 115 healthy blood donors (aged 18 to 75 years) from Zhejiang Hospital (Zhejiang, China) with no history of TB, hepatitis B, AIDS, or other diseases were also included in the study.

Plasma samples were collected in heparin lithium-anticoagulant tubes and centrifuged at 3000 rpm at 4 °C for 10 min. Samples were dispensed into sterile centrifuge tubes and stored at −80 °C. Data such as lipoprotein-a, apolipoprotein-A1, apolipoprotein-B, total cholesterol (TC), high-density lipoprotein (HDL), low-density lipoprotein (LDL), and triglycerides (TG) levels were recorded for PYD, HFYD, and DQY cases and healthy controls. Differences were analyzed by one-way ANOVA followed by Tukey’s post-hoc test, *χ* ^2^ test, or unpaired *t*-test using GraphPad Prism 5 (GraphPad Software, Inc., USA) and one-sample *t*-test after taking the logarithm using SPSS 16.0.

### Chemicals and reagents

TRIzol® reagent was purchased from Invitrogen Life Technologies, and the RNeasy Mini Kit was obtained from Qiagen (Valencia, CA, USA). The Quick Amp Labeling Kit (One-Color), gene expression hybridization kit, gene expression wash buffer, and microarray scanner were obtained from Agilent (California, USA). The magnetic stir plate was obtained from Corning Incorporated (New York, USA).

### RNA isolation

Eleven PYD cases, 11 HFYD cases, 11 DQY cases, and 11 healthy controls were randomly chosen for the following experiments. Each experimental group was divided into three biological repeats. Plasma (200 μL) from each specimen was used to extract total RNA with TRIzol reagent (Invitrogen Life Technologies), and total RNA was eluted in 85 μL of RNase-free water. An RNeasy Mini Kit (Qiagen p/n 74104) was used to purify total RNA according to the manufacturer’s instructions. RNA quantity and concentration were evaluated using a NanoDrop ND-1000 spectrophotometer at an absorbance ratio of A260/A280. The nucleic acid was considered pure when the absorbance ratio was 1.8–2.0.

### DNA microarray

The Human LncRNA Microarray V3.0 (Arraystar Co. USA) allows the global profiling of human lncRNAs and protein-coding transcripts. An estimated 30,586 lncRNAs were constructed using the most highly respected public transcriptome databases, including Refseq, Gencode, and UCSC known genes, and the lncRNA microarray can detect 26,109 coding transcripts. A specific exon or splice junction probe accurately identified each transcript. Negative probes and positive probes (housekeeping genes) were also printed onto the array for hybridization quality control [26].

### RNA labeling and array hybridization

Total RNA (1 μg) from each group was amplified and transcribed into cyanine 3-labeled cRNA according to the instructions for the Quick Amp Labeling Kit, One-Color (Agilent). The labeled cRNAs were purified, and the concentration and specific activity (pmol Cy3/μg cRNA) were measured using a NanoDrop ND-1000 spectrophotometer. Hybridization was performed using an Agilent Gene Expression Hybridization Kit according to the manufacturer’s guidelines. Briefly, final 1× blocking agent and 1× fragmentation buffer were added to the labeled cRNA and incubated at 60 °C to fragment RNA for 30 min. GE Hybridization Buffer HI-RPM was mixed with the samples to stop the fragmentation reaction. A gasket slide was loaded into the Agilent SureHyb chamber before the hybridization samples were dispensed into the gasket well, and the Human LncRNA Array V3.0 slide was assembled. The slides were hybridized in a hybridization oven at 65 °C for 17 h, washed with Gene Expression Wash Buffer, fixed and immediately scanned in an Agilent Microarray Scanner (Agilent p/n G2565BA) [27].

### Data analysis

The array images were analyzed using Agilent Feature Extraction (version 11.0.1.1) software, and subsequent quantile normalization and further data analyses were performed in the GeneSpring GX v11.5.1 package (Agilent Technologies). LncRNAs and mRNAs flagged as Present or Marginal (“All Targets Value”) in at least three of 12 samples were chosen for further data analyses to remove transcripts with unreliable expression. Significantly differentially expressed lncRNAs and mRNAs between the two groups were identified using Volcano Plot filtering, and the expression patterns were analyzed using hierarchical clustering [28].

### lncRNA classification and pathway analysis

To explore potential functional relationship between lncRNAs and related coding genes, significantly expressed ncRNAs were classified into different subgroups, including enhancer lncRNAs near coding genes, enhancer lncRNA profiling, homeobox transcription factor (HOX) cluster profiling, long intergenic noncoding RNAs (lincRNAs) near coding genes, and lincRNA profiling. Pathway analyses of differentially expressed mRNAs were performed using the Kyoto Encyclopedia of Genes and Genomes (KEGG) database. Gene ontology (GO) was analyzed online (http://www.geneontology.org) to determine the broad attributes of genes and gene products, which were classified into three domains: biological process, cellular component, and molecular function. The overlap between differentially expressed genes and GO annotation was also analyzed using Fisher’s exact test [28].

## Results

### Clinical characteristics of TB cases with different TCM syndromes

**Fig. 1:**
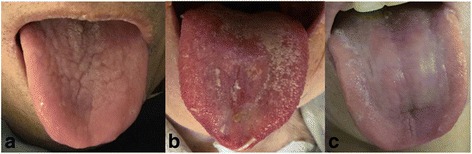
Tongue manifestations of TB patients. **a** PYD syndrome of TB (red tongue with thin fur). **b** HFYD syndrome of TB (red or dark red tongue with thin yellow or eroded fur). **c** DQY syndrome of TB (red and tender tongue with thin fur)

PYD patients exhibited the following clinical symptoms and signs: tussiculation; scant sticky and white sputum or blood-stained sputum; dry mouth and pharynx; red tongue with thin fur; and weak and rapid pulse (Fig. 1a).

HFYD patients exhibited the following clinical characteristics: cough and breathlessness; hemoptysis; scant sticky sputum with white or yellow color; dry mouth and pharynx; red cheeks in the afternoon; tidal fever; steaming sensation in the bone; night sweats; red or dark red tongue with thin yellow or eroded fur; and weak and rapid pulse (Fig. 1b).

DQY patients exhibited the following clinical symptoms: cough with shortness of breath; clear and thin sputum; hemoptysis; physical and mental fatigue; spontaneous perspiration and night sweats; abdominal distension; anorexia; loose stool; red, tender tongue with thin fur; and weak and rapid pulse (Fig. 1c).

### Statistical analysis of clinical indexes in TB cases

Statistical analyses of clinical indexes were performed between 92 PYD cases (aged 40.13 ± 16.16 years), 124 HFYD cases (aged 40.85 ± 15.85 years), 76 DQY cases (aged 43.95 ± 13.49 years), and 115 healthy controls (aged 39.30 ± 9.60 years). There were no significant differences in gender or age between the four groups. However, one-way ANOVA showed that the cholesterol levels of TB patients were significantly different compared to the healthy controls. The TC values in PYD cases, HFYD cases, DQY cases, and healthy controls were 3.83 ± 1.06 mmol/L, 3.85 ± 0.80 mmol/L, 3.79 ± 0.87 mmol/L, and 4.65 ± 0.52 mmol/L, respectively (*P* < 0.0001). HDL values were 1.05 ± 0.36 mmol/L, 1.10 ± 0.40 mmol/L, 1.04 ± 0.35 mmol/L, and 1.44 ± 0.26 mmol/L, respectively (*P* < 0.0001). LDL values were 2.74 ± 0.52 mmol/L, 2.31 ± 0.66 mmol/L, 2.30 ± 0.72 mmol/L, and 2.73 ± 0.52 mmol/L, respectively (*P* = 0.0002). TG values were 1.08 ± 0.57 mmol/L, 0.97 ± 0.41 mmol/L, 1.20 ± 0.89 mmol/L, and 0.95 ± 0.33 mmol/L, respectively (*P* = 0.0085). The TG value was significantly higher in DQY cases than in PYD and DQY cases (*P* = 0.0231, Table 1). One-sample *t*-tests were also performed to analyze differences between TB patients with different TCM syndromes and normal reference ranges after taking the logarithm. Various significant differences between TB patients with different TCM syndromes and normal reference values of blood fat indexes were observed (Additional file 1).

**Table 1:** Characteristics of TB cases with PYD, HFYD and DQY syndromes

	Healthy Controls (*N* = 115)	PYD (*N* = 92)	HFYD (*N* = 124)	DQY (*N* = 76)	*P* value
Age, age range (Mean ± SD)	39.30 ± 9.60	40.13 ± 16.16	40.85 ± 15.85	43.95 ± 13.49	0.1409^a^
Gender (female: male)	58/57	40/52	50/74	28/48	0.6558^b^
Abnormal chest radiograph (X, CT), no. (%)	ND	92(100)	124(100)	76(100)	/
Positive sputum smears, no. (%)	ND	77(84.00)	100(81)	56(74.00)	/
Lipoprotein a (mg/L)	ND	261.41 ± 279.31	196.81 ± 189.58	264.72 ± 242.90	/
Apolipoprotein A1 (apoA1) (g/L)	ND	1.05 ± 0.24	1.11 ± 0.28	1.07 ± 0.22	/
Apolipoprotein B (apoB) (g/L)	ND	0.78 ± 0.24	0.81 ± 0.20	0.76 ± 0.17	/
Total cholesterol (TC) (mmol/L)	4.65 ± 0.52	3.83 ± 1.06	3.85 ± 0.80	3.79 ± 0.87	<0.0001^*** a^
High-density lipoprotein (HDL) (mmol/L)	1.44 ± 0.26	1.05 ± 0.36	1.10 ± 0.40	1.04 ± 0.35	<0.0001^*** a^
Low-density lipoprotein (LDL) (mmol/L)	2.74 ± 0.52	2.43 ± 1.20	2.31 ± 0.66	2.30 ± 0.72	0.0002^*** a^
Triglyceride (TG) (mmol/L)	0.95 ± 0.33	1.08 ± 0.57	0.97 ± 0.41	1.20 ± 0.89	0.0085^** a^
		1.02 ± 0.49		1.20 ± 0.89	0.0252^* c^

There were no significant differences in gender and age between healthy controls and TB cases with PYD, HFYD and DQY syndromes. However, the cholesterol levels of TB patients, such as TC, HDL, LDL, and TG, were significantly different from those of healthy controls

^a^ *P*-value between healthy controls and TB cases with PYD, HFYD and DQY syndromes for one-way ANOVA followed by Tukey’s post-hoc test

^b^ *P*-value between healthy controls and TB cases with PYD, HFYD and DQY syndromes for the χ^2^ test

^c^ *P*-value between HFYD cases and other TB patients for the unpaired *t*-test

*N* number of subjects, *ND* not determined. *P < 0.05. **P < 0.01. *** *P* < 0.001

### lncRNA microarray profiling of TB cases with different TCM syndromes

**Fig. 2:**
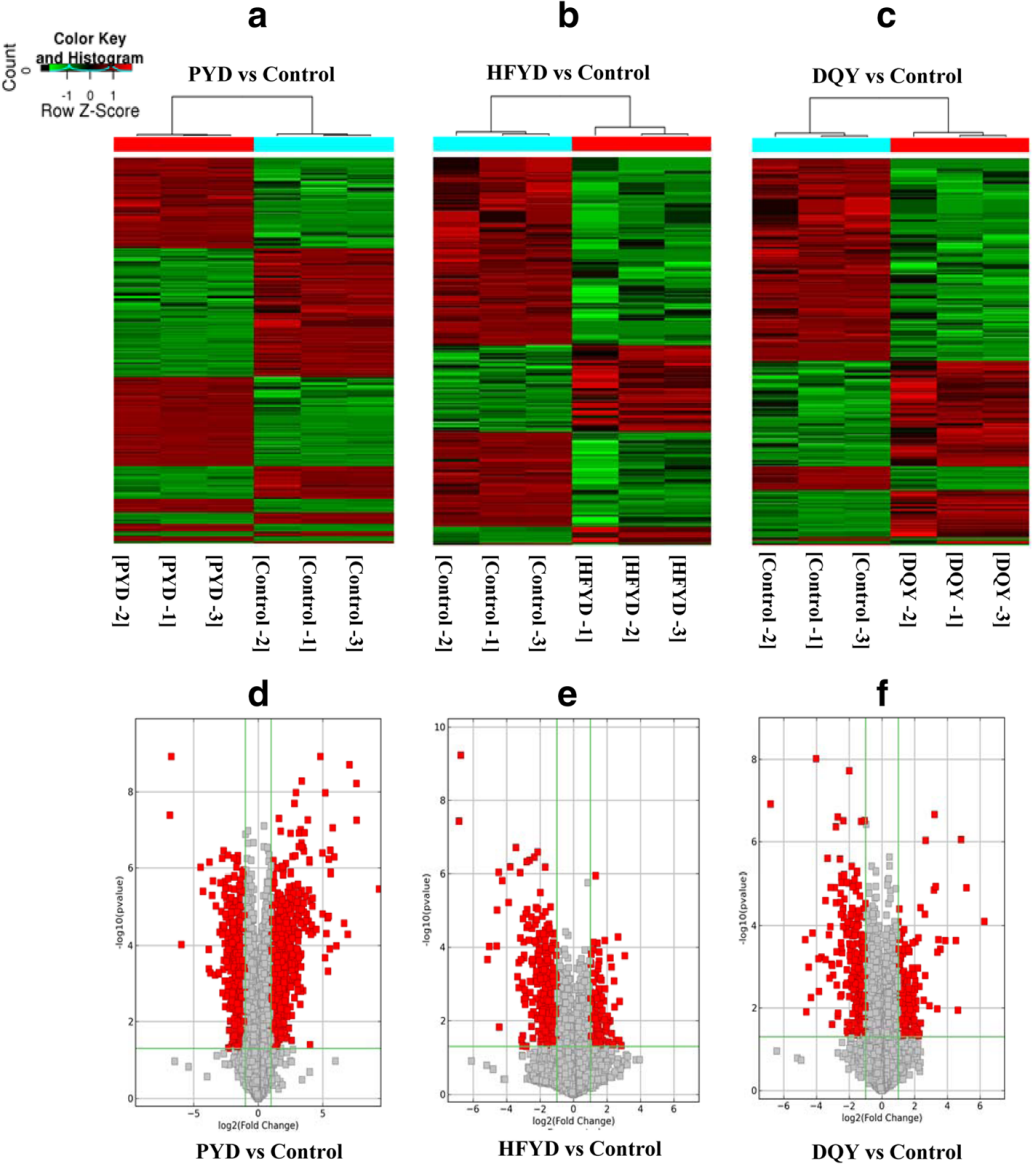
lncRNA volcano plots and hierarchical clustering dendrogram of TB patients with distinctive TCM syndromes. **a**-**c** lncRNA volcano plots (fold change >2.0 and *P* < 0.05). The vertical lines correspond to 2.0-fold up and down (log2 ratio), and the horizontal line represents a *P*-value of 0.05. Each point in the plot represents a different transcript. Transcripts are isolated based on statistical significance and magnitude of change. Red squares represent significantly differentially expressed lncRNAs. **d–f** Hierarchical clustering dendrogram representing distinguishable lncRNA expression profiling of samples. PYD: pulmonary Yin deficiency syndrome; HFYD: hyperactivity of fire due to Yin deficiency syndrome; DQY: deficiency of Qi and Yin syndrome

The distribution of samples for microarray detection is shown in Additional file 2. Microarray profiling of 30,586 lncRNAs was analyzed using Arraystar Human LncRNA Microarray V3.0, and lncRNAs with fold changes >2.00 and *P* < 0.05 were considered significantly different. A total of 566 differentially expressed lncRNAs were identified in PYD cases, including 347 up-regulated and 219 down-regulated lncRNAs. Fifty-five differentially expressed lncRNAs were identified in HFYD cases, including 31 up-regulated and 24 down-regulated lncRNAs. Sixty differentially expressed lncRNAs were identified in DQY cases, including 35 up-regulated and 25 down-regulated lncRNAs. Most of the differentially expressed lncRNAs were intergenic lncRNAs (61.34%), natural antisense lncRNAs (14.63%), or intronic antisense lncRNAs (11.49%). The remainder were exon sense-overlapping lncRNAs, intron sense-overlapping lncRNAs, and bidirectional lncRNAs. Figure 2 shows the volcano plots and hierarchical clustering of the differentially expressed lncRNAs.

### mRNA microarray profiling of TB cases with different TCM syndromes

**Fig. 3:**
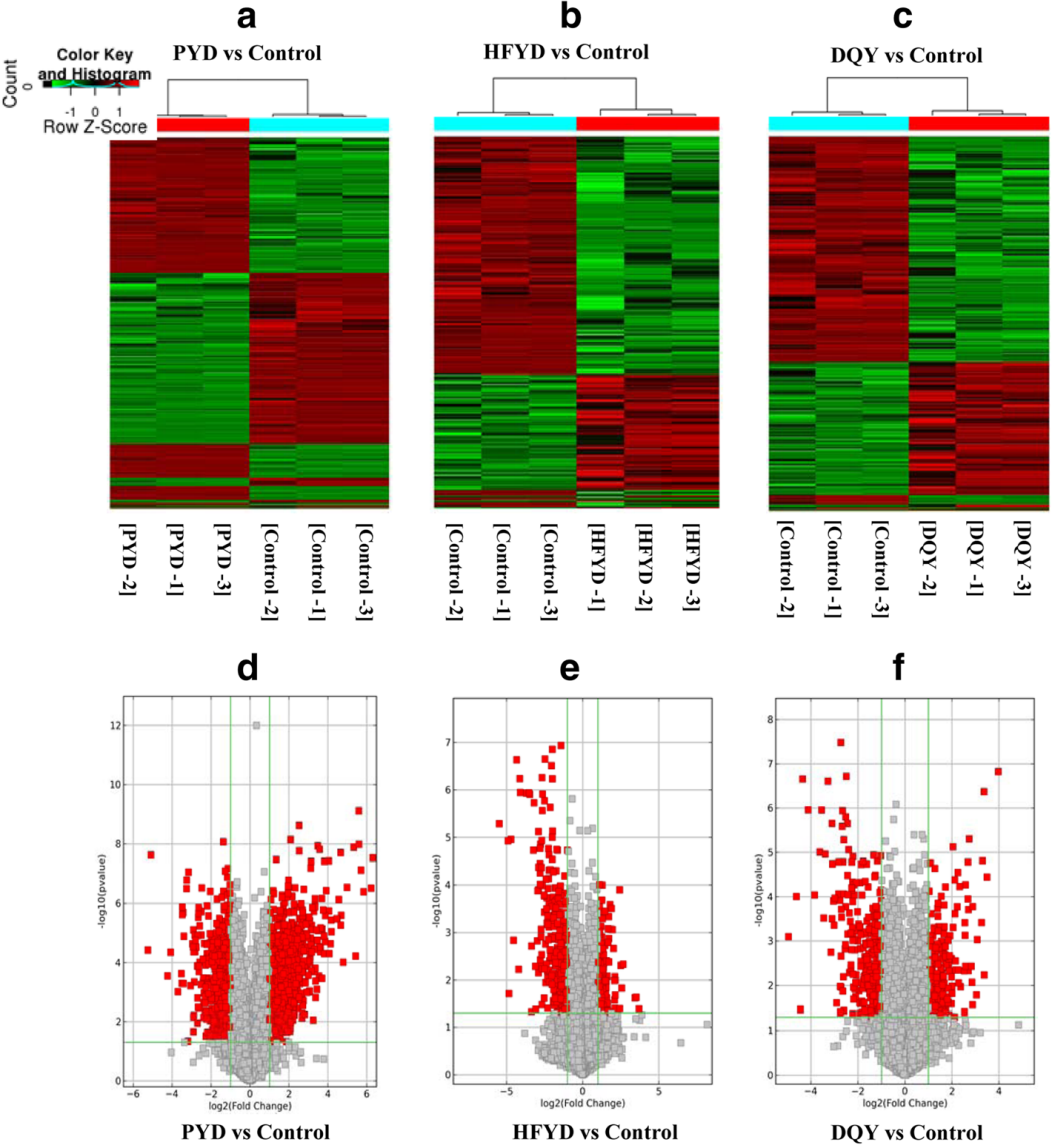
mRNA volcano plots and hierarchical clustering dendrogram of TB patients with distinctive TCM syndromes. **a**–**c** mRNA volcano plots (fold change >2.0 and *P* < 0.05). The vertical lines correspond to 2.0-fold up and down (log2 ratio), and the horizontal line represents a *P*-value of 0.05. Each point in the plot represents a different transcript. Transcripts are isolated based on statistical significance and magnitude of change. Red squares represent significantly differentially expressed mRNAs. **d–f** Hierarchical clustering dendrogram representing distinguishable mRNA expression profiling of the samples. PYD: pulmonary Yin deficiency syndrome; HFYD: hyperactivity of fire due to Yin deficiency syndrome; DQY: deficiency of Qi and Yin syndrome

Microarray profiling of 26,109 mRNAs was also performed using Arraystar Human LncRNA Microarray V3.0. A total of 634 differentially expressed mRNAs were identified in PYD patients, including 404 up-regulated and 230 down-regulated mRNAs. Forty-seven differentially expressed mRNAs were identified in HFYD patients, including 28 up-regulated and 19 down-regulated mRNAs. Sixty-three differentially expressed mRNAs were identified in DQY patients, including 44 up-regulated and 19 down-regulated mRNAs. The volcano plots and hierarchical clustering of differentially expressed mRNAs are shown in Fig. 3.

### Biological analysis

**Fig. 4:**
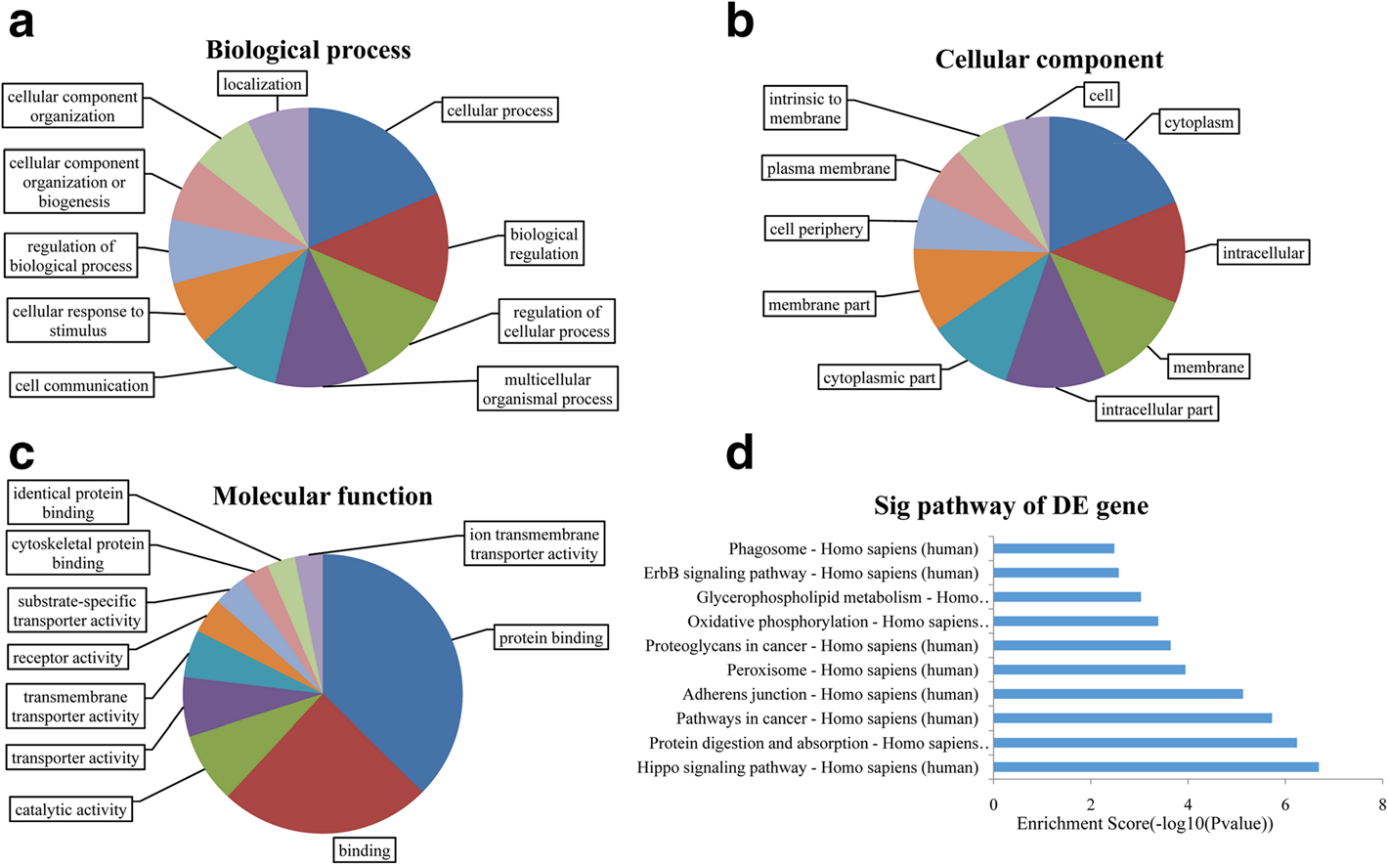
Gene ontology and pathway analyses of differentially expressed transcripts

GO analysis was performed to determine the functions of genes and gene products involved in biological processes, cellular components and molecular functions. Fisher’s exact test was performed to determine the overlap between the differentially expressed list and the GO annotation. The significance of GO term enrichment among the differentially expressed genes was shown using *P* values. The highest enriched GO terms among differentially expressed transcripts between TB syndromes were cellular process (GO: 0009987; Ontology: biological process, *P* = 4.23124E-05) (Fig. 4a), cytoplasm (GO: 0005737; Ontology: cellular component, *P* = 0.000754106) (Fig. 4b), and protein binding (GO: 0005515; Ontology: molecular function, *P* = 0.001293641) (Fig. 4c).

Pathway analysis indicated that 61 pathways were associated with differentially expressed transcripts. The most enriched pathway was “Hippo signaling pathway-Homo sapiens (human)” (*P* = 0.000164), which was composed of 19 differentially expressed genes, and “Protein digestion and absorption-Homo sapiens (human)” (*P* = 5.89017E-05), which was also composed of 19 differentially expressed genes (Fig. 4d). Seven transcripts (*COL4A6, PGA3, PGA4, PGA5, SLC1A5, SLC7A8, and SLC9A3*) involved in pathways of protein digestion and absorption were up-regulated in PYD patients compared to HFYD and DQY patients. Sixteen transcripts linked to oxidative phosphorylation pathways were up-regulated in HFYD patients compared to the healthy controls, and two of these transcripts (*CYC1 and PPA2*) were up-regulated in HFYD patients compared to PYD and DQY patients. However, two transcripts (*ACOT1 and PTPLA*) related to the fatty acid elongation pathway were up-regulated in DQY patients compared to the healthy controls. We screened 20 differentially expressed mRNAs and lncRNAs in different TCM syndromes of TB based on the fold changes and the results of statistical analyses (Tables 2, 3 and 4).

**Table 2:** Differentially expressed lncRNAs and mRNAs in TB cases with PYD syndrome

Seqname	GeneSymbol	*P*-value			Absolute Fold Change			Associated Gene
		A vs. control	A vs. B	A vs. C	A vs. control	A vs. B	A vs. C	
lncRNA								
ENST00000536029	RP11-392P7.8	3.41E-07	8.50E-04	1.35E-04	47.87	44.37	44.85	GPRC5D
ENST00000543515	RP11-392P7.8	4.86E-05	1.72E-05	1.43E-04	39.01	44.01	31.53	GPRC5D
NR_036524	MICA	1.70E-05	1.20E-04	4.19E-05	44.00	36.07	29.72	MICA
ENST00000467458	RP11-796I2.2	1.48E-05	4.18E-07	1.91E-05	31.88	49.41	43.14	PRKAG2
uc002mej.1	AK056073	2.89E-06	1.40E-07	1.16E-07	5.28	5.46	5.52	ACSBG2
TCONS_00002486	XLOC_001036	5.51E-06	1.81E-06	7.44E-04	−5.57	−5.62	−4.58	KCNN3
DA401339	/	1.76E-04	8.76E-04	4.92E-04	−3.65	−4.94	−5.47	POU3F2
NR_036569	PILRB	1.73E-05	3.32E-05	1.42E-04	−4.32	−3.77	−4.53	PILRB
ENST00000458682	LINC00202	1.72E-03	1.94E-03	3.23E-03	−5.12	−5.48	−4.45	/
ENST00000517670	RP11-363E6.3	5.20E-05	9.24E-03	6.41E-04	−2.66	−2.75	−2.74	FABP5
mRNA								
NM_033641	COL4A6	6.59E-04	1.23E-02	3.81E-05	3.04	3.63	2.76	/
NM_001079807	PGA3	1.32E-05	1.62E-03	9.73E-06	2.71	2.41	2.86	/
NM_001079808	PGA4	7.14E-06	2.51E-03	9.10E-05	5.07	5.30	4.12	/
NM_014224	PGA5	2.76E-06	2.84E-04	1.83E-02	2.88	4.08	2.81	/
ENST00000412532	SLC1A5	5.62E-03	2.18E-03	8.24E-04	4.78	11.40	8.89	/
NM_012244	SLC7A8	6.75E-05	2.77E-03	1.52E-03	4.45	4.81	3.47	/
NM_004174	SLC9A3	5.38E-06	5.64E-06	5.33E-06	4.85	4.81	4.86	/
NM_199235	COLEC11	3.00E-05	6.87E-04	5.33E-03	−6.77	−2.80	−4.07	/
NM_020664	DECR2	1.37E-05	7.59E-05	7.84E-04	−3.54	−3.61	−3.76	/
NM_018248	NEIL3	2.06E-04	8.97E-05	9.04E-05	−2.97	−2.97	−3.28	/

Twenty of the most significantly differentially expressed lncRNAs and mRNAs in TB cases with PYD syndrome compared to healthy controls and TB cases with HFYD syndrome and DQY syndrome

Fold change >2: up-regulated; fold change < -2: down-regulated

A: PYD syndrome; B: HFYD syndrome; C: DQY syndrome

**Table 3:** Differentially expressed lncRNAs and mRNAs in TB cases with HFYD syndrome

Seqname	GeneSymbol	*P*-value			Absolute Fold Change			Associated Gene
		B vs. control	B vs. A	B vs. C	B vs. control	B vs. A	B vs. C	
lncRNA								
TCONS_00008415	XLOC_003867	4.72E-02	1.71E-02	3.09E-02	7.24	14.65	10.20	/
NR_024499	FMR1-AS1	3.45E-02	2.96E-02	3.62E-02	6.64	8.06	7.25	FMR1
ENST00000510610	RP11-706C16.8	1.95E-04	1.72E-05	2.80E-05	4.57	6.58	6.39	LY6D
ENST00000521600	PVT1	8.03E-03	1.69E-03	1.88E-02	4.11	5.83	4.95	/
ENST00000448365	AC078842.4	6.88E-03	3.39E-03	2.75E-03	3.09	3.25	4.11	PTN
uc021pjm.1	HM358976	2.11E-04	5.85E-08	4.50E-05	−5.46	−4.76	−5.48	/
ENST00000441029	RP4-553 F4.2	4.27E-05	9.12E-04	4.26E-04	−3.49	−3.27	−4.65	ZNF341
ENST00000444112	AC005808.3	4.42E-03	2.13E-03	2.58E-02	−2.66	−3.30	−3.08	ZNF217
TCONS_00004246	XLOC_002087	7.60E-05	1.39E-03	3.01E-04	−4.72	−3.12	−3.88	/
ENST00000434072	GNG12-AS1	1.72E-03	1.54E-04	1.60E-03	−2.96	−6.46	−2.62	WLS
mRNA								
NM_001916	CYC1	1.26E-04	1.33E-03	1.67E-04	5.39	5.61	7.44	/
NM_176869	PPA2	1.44E-02	1.67E-02	4.48E-02	2.70	2.02	2.06	/
ENST00000418434	CASP5	2.61E-02	3.75E-02	1.67E-02	4.29	3.82	5.21	/
NM_182609	ZNF677	8.52E-03	2.47E-02	2.56E-03	4.35	3.61	5.60	/
NM_001135086	PRSS41	3.90E-02	1.15E-02	4.07E-02	4.34	9.42	4.54	/
NM_207174	ABCG1	4.65E-03	2.59E-03	3.68E-02	6.35	8.32	4.40	/
NM_001101	ACTB	1.95E-03	3.10E-04	1.99E-03	−2.45	−4.22	−3.09	/
NM_001135575	C6orf228	1.23E-03	1.10E-03	3.89E-03	−6.66	−7.04	−5.13	/
NM_001166415	EHHADH	3.06E-02	2.25E-02	1.93E-02	−2.79	−2.87	−3.15	/
NM_152496	MANEAL	2.40E-02	1.28E-02	1.36E-02	−5.78	−8.54	−8.88	/

Twenty of the most significantly differentially expressed lncRNAs and mRNA in TB cases with HFYD syndrome compared to healthy controls and TB cases with PYD syndrome and DQY syndrome

Fold change >2: up-regulated; fold change < -2: down-regulated

A: PYD syndrome; B: HFYD syndrome; C: DQY syndrome

**Table 4:** Differentially expressed lncRNAs and mRNAs in TB cases with DQY syndrome

Seqname	Gene Symbol	*s*-value			Absolute Fold Change			Associated Gene
		C vs. control	C vs. A	C vs. B	C vs. control	C vs. A	C vs. B	
lncRNA								
ENST00000412526	LINC00161	1.07E-03	1.72E-06	7.63E-04	4.59	6.66	4.13	/
uc004erg.1	BC028211	2.34E-02	4.18E-02	1.53E-02	5.21	4.40	7.54	SLC25A5
NR_036516	C17orf62	1.13E-05	1.19E-05	2.03E-04	9.79	9.70	7.45	C17orf62
ENST00000434601	LINC00422	1.05E-02	1.62E-02	2.06E-02	10.46	7.46	6.49	/
ENST00000522460	RP1-84O15.2	8.86E-03	9.02E-03	9.01E-03	7.49	7.42	7.42	/
ENST00000452110	PI4KAP1	1.63E-02	8.34E-05	8.64E-03	−2.42	−8.92	5.27	/
BM728564	/	1.88E-03	5.54E-03	4.47E-02	−9.20	−5.69	3.54	/
ENST00000528696	CTD-3076O17.1	1.88E-03	4.12E-02	1.57E-02	−2.15	−4.07	−2.09	ADAMTS17
ENST00000582033	CTD-2124B20.2	9.98E-03	1.21E-04	1.86E-04	−2.81	−4.08	−2.39	/
TCONS_00012029	XLOC_005561	9.25E-05	3.03E-02	1.68E-04	−2.90	−3.51	−2.29	/
mRNA								
NM_013322	SNX10	2.73E-02	2.72E-02	2.99E-02	7.36	7.39	6.99	/
NM_004436	ENSA	9.27E-04	9.47E-04	4.01E-03	8.45	8.36	6.76	/
NM_153182	MINA	5.81E-03	5.91E-03	5.90E-03	10.37	10.27	10.28	/
NM_014178	STXBP6	1.50E-07	1.83E-07	1.59E-07	15.71	15.56	15.56	/
NM_183375	PRSS48	3.58E-05	1.94E-06	1.27E-06	11.26	11.92	12.46	/
NM_001037161	ACOT1	2.39E-02	7.52E-02	3.50E-01	2.15	1.68	1.67	/
NM_014241	PTPLA	2.25E-02	7.65E-01	5.54E-02	3.11	1.05	2.17	/
NM_001040443	PHF11	1.82E-02	1.01E-02	2.71E-02	−2.71	−3.85	−2.93	/
NM_001099455	CPPED1	1.21E-03	2.01E-03	2.32E-02	−5.78	−4.58	−2.25	/
NM_080627	SOGA1	7.61E-04	2.65E-04	4.96E-04	−2.48	−4.33	−3.32	/

Twenty of the most significantly differentially expressed lncRNAs and mRNA in TB cases with DQY syndrome compared to healthy control s and TB cases with PYD syndrome and HFYD syndrome

Fold change >2: up-regulated; fold change < -2: down-regulated

A: PYD syndrome; B: HFYD syndrome; C: DQY syndrome

## Discussion

TCM states that disease occurs when Yin and Yang are unbalanced or the flow of Qi and blood is disturbed [15]. The major causes of TB are infection by Mtb and Yin deficiency [29]. Consumption of lung-Yin in the early stage of TB causes pulmonary Yin deficiency syndrome (PYD). Hyperactivity of liver-fire occurs with the development of lung-Yin consumption and leads to hyperactivity of fire due to Yin deficiency syndrome (HFYD). Harmony of Qi and blood is disturbed in some chronic TB patients and may cause a deficiency of Qi and Yin syndrome (DQY) [29, 30]. There is a lack of research on the subtle changes between different TCM syndromes, which is the major challenge in the interpretation of the theories of TCM using traditional methods [15, 17, 29]. We previously investigated the proteomic profiles of TB cases with TCM syndromes using SELDI-TOF MS and iTRAQ-2DLC-MS/MS and identified several differentially expressed serum proteins in PYD, HFYD, and DQY cases [10, 31]. Thus, subtle changes between different TCM syndromes of TB were reflected in serum proteomics, suggesting that subtle changes in PYD, HFYD and DQY cases may also be reflected in transcriptomics.

In the current study, more differentially expressed mRNAs and lncRNAs were detected in PYD patients than HFYD and DQY patients. A total of 634 mRNAs and 566 lncRNAs were differentially expressed in PYD patients. However, 47 mRNAs and 55 lncRNAs were differentially expressed in HFYD patients, and 63 mRNAs and 60 lncRNAs were differentially expressed in DQY patients. These results indicate that more abnormal gene expression occurred in PYD patients.

A total of 19 mRNAs involved in the pathway of protein digestion and absorption were differentially expressed in PYD cases compared to the healthy controls, and seven transcripts (*COL4A6*, *PGA3*, *PGA4*, *PGA5*, *SLC1A5*, *SLC7A8*, and *SLC9A3*) were also up-regulated in PYD cases compared to HFYD and DQY cases. *COL4A6* encodes the alpha-6 chain of type IV collagen of basal membranes and is related to the prognosis of esophageal squamous cell carcinoma [32]. *PGA3*, *PGA4*, and *PGA5* encode pepsinogen A (PGA), and the differential expression of *PGA3*, *PGA4*, and *PGA5* is related to the pre-neoplastic nature in patients with Barrett’s esophagus [33]. *SLC7A8* and *SLC9A3* are solute carrier family genes, and the product of *SLC7A8* participates in the transport of amino acids [34]. *SLC9A3* encodes Na(+)/H(+) exchanger (NHE3), which is down-regulated in patients with ulcerative colitis [35]. TCM considers the spleen the ‘mother organ’ to the lungs, and thus the spleen may be similarly affected by conditions that affect the ‘child organ’ [36]. PYD syndrome often leads to spleen deficiency, and the clinical symptoms of PYD patients with spleen deficiency were anorexia, poor appetite, and loose stools [36]. Patients with spleen deficiency are generally characterized by digestive system disorders [37]. Therefore, we suspect that differentially expressed mRNAs (*COL4A6*, *PGA3*, *PGA4*, *PGA5*, *SLC1A5*, *SLC7A8*, and *SLC9A3*) involved in the protein digestion and absorption pathway may be related to the spleen deficiency in PYD patients.

The expression of 16 mRNAs involved in the pathway of oxidative phosphorylation was significantly increased in HFYD patients compared to the healthy controls, and two mRNAs (*CYC1*, *PPA2*) were also up-regulated. *CYC1* encodes the cytochrome *c*1 subunit of respiratory chain complex III, which mediates the transfer of electrons from cytochrome *b* to cytochrome *c* during oxidative phosphorylation [38–40]. The *PPA2* gene encodes mitochondrial pyrophosphatase 2, which catalyzes the hydrolysis of pyrophosphate to generate inorganic phosphate in cellular enzymatic reactions [41]. *PPA2* is required for the maintenance of mitochondrial DNA and the synthesis of DNA, RNA, cAMP, and cGMP [41]. Oxidative phosphorylation is the major source of ATP and energy production, and mitochondria are the primary site of oxidative phosphorylation reactions [42]. Pulmonary TB is a typical consumptive disease with symptoms of weight loss, energy expenditure, and fat reduction [43]. Therefore, increased expression of *CYC1* and *PPA2* mRNAs in the pathway of oxidative phosphorylation may be linked to the energy consumption observed in HFYD syndrome.

Two mRNAs (*ACOT1*, *PTPLA*) involved in the fatty acid elongation pathway were up-regulated in DQY patients compared to the healthy controls. *ACOT1* encodes acyl-CoA thioesterase 1, which hydrolyzes acyl-CoAs into free fatty acids and CoASH, thereby regulating intracellular levels of free fatty acids and CoASH [44]. *PTPLA* (also known as *HACD1*) encodes 3-hydroxyacyl-CoA dehydratase 1, which affects the third step (dehydration) in the elongation of very long chain fatty acids and is necessary for muscle function [45]. Clinical analyses revealed that cholesterol levels (TG, TC, HDL, LDL) differed significantly between TB patients and healthy controls, and the value of TG was significantly increased in DQY patients compared to PYD and DQY patients (Table 1). The hydrolysis of triglyceride (fat) from TB patients is necessary for the survival and virulence of *Mycobacterium tuberculosis* [46, 47]. Therefore, the up-regulated *ACOT1* and *PTPLA* mRNAs linked to the fatty acid elongation pathway may be associated with the abnormality of blood fats in DQY syndrome.

Differentially expressed lncRNAs were identified in PYD, HFYD, and DQY patients. Ten differentially expressed lncRNAs for each TCM syndrome of TB were screened based on fold changes. G-protein coupled receptor family C group 5 member D, MHC class I polypeptide-related sequence A isoform 2,5’-AMP-activated protein kinase subunit gamma-2 isoform a, and paired immunoglobulin-like type 2 receptor beta precursor were the associated proteins of differentially expressed lncRNAs in PYD cases. Lymphocyte antigen 6D precursor, pleiotrophin precursor, and zinc finger protein were the associated proteins of differentially expressed lncRNAs in HFYD patients. ADP/ATP translocase 2 and a disintegrin and metalloproteinase with thrombospondin motifs 17 preproprotein were the associated proteins of differentially expressed lncRNAs in DQY patients. However, the functions of most lncRNAs have not been reported. Therefore, the differentially expressed lncRNAs in PYD, HFYD, and DQY patients and their functional relationship with the subtle changes between different TCM syndromes of TB requires further investigation.

## Conclusion

This study revealed significantly altered lncRNA and mRNA expression profiles in the PYD, HFYD and DQY syndromes of TB. The pathway enrichment of differentially expressed transcripts was also analyzed using bioinformatics methods. The enhanced expression of mRNAs involved in the protein digestion and absorption pathway in PYD patients may be related to the spleen deficiency in PYD syndrome. The increased expression of *CYC1* and *PPA2* mRNAs in the oxidative phosphorylation pathway may be linked to the energy consumption in HFYD syndrome. The up-regulated *ACOT1* and *PTPLA* mRNAs linked to the fatty acid elongation pathway may be associated with the abnormality of blood fats in DQY syndrome. These results indicated that the expression profile analysis of lncRNAs and mRNAs may be a novel method to explore the biological essence of TCM syndromes. However, the functional roles of lncRNAs and mRNAs in different TCM syndromes of TB require further investigation.

## Additional files

Additional file 1: Clinical data for TB cases with PYD, HFYD and DQY syndromes and normal reference ranges. *P* values between TB cases with PYD, HFYD and DQY syndromes and the normal reference range were determined by one-sample *t*-test after taking the logarithm and comparison to the median. **P* < 0.05. ***P* < 0.01. *** *P* < 0.001 (DOCX 17 kb)
Additional file 2: Sample data for TB PYD, HFYD, and DQY patients and healthy controls. (DOCX 15 kb)

## Abbreviations

DQY: Deficiency of Qi and Yin
GO: Gene ontology
HDL: High-density lipoprotein
HFYD: Hyperactivity of fire due to Yin deficiency
HOX: Homeobox transcription factors
KEGG: Kyoto encyclopedia of genes and genomes
LDL: Low-density lipoprotein
lincRNAs: long intergenic noncoding RNAs
lncRNAs: Long non-coding RNAs
Mtb: *Mycobacterium tuberculosis*
PYD: Pulmonary Yin deficiency
TB: Tuberculosis
TC: Total cholesterol
TCM: Traditional Chinese medicine
TG: Triglyceride
