# Supplementary material for: Microarray expression profile analysis of mRNAs and long non-coding RNAs in pulmonary tuberculosis with different traditional Chinese medicine syndromes
Source: BMC Complement Altern Med. 2016 Nov 17;16:472. doi: 10.1186/s12906-016-1436-y (PMC5114807; doi:10.1186/s12906-016-1436-y)
Supplement: Additional file 1: — Clinical data for TB cases with PYD, HFYD and DQY syndromes and normal reference ranges. P values between TB cases with PYD, HFYD and DQY syndromes and the normal reference range were determined by one-sample t-test after taking the logarithm and comparison to the median. *P < 0.05. **P < 0.01. *** P < 0.001 (DOCX 17 kb) [file 12906_2016_1436_MOESM1_ESM.docx]

**Additional File 1**. Clinical data for TB cases with PYD, HFYD and DQY syndromes and normal reference ranges.

|  | **Reference Values** | **PYD (N=92)** | ***P* value** | **HFYD (N=124)** | ***P* value** | **DQY (N=76)** | ***P* value** |
| --- | --- | --- | --- | --- | --- | --- | --- |
| Age, age range (Mean ± SD) | / | 40.13±16.16 | / | 40.85±15.85 | / | 43.95±13.49 | / |
| Gender (female: male) | / | 40/52 | / | 50/74 | / | 28/48 | / |
| Abnormal chest radiograph (X, CT), no. (%) | / | 92(100) | / | 124(100) | / | 76(100) | / |
| Positive sputum smears, no. (%) | / | 77(84.00) | / | 100(81) | / | 56(74.00) | / |
| Lipoprotein a (mg/L) | 0-300.00 | 261.41±279.31 | 0.0080^**^ | 196.81±189.58 | 0.0350^*^ | 264.72±242.90 | 0.0040^**^ |
| Apolipoprotein A1 (apoA1) (g/L) | 1.20-1.60 | 1.05±0.24 | < 0.0001^***^ | 1.11±0.28 | < 0.0001^***^ | 1.07±0.22 | < 0.0001^***^ |
| Apolipoprotein B(apoB) (g/L) | 0.80-1.05 | 0.78±0.24 | < 0.0001^***^ | 0.81±0.20 | < 0.0001^***^ | 0.76±0.17 | < 0.0001^***^ |
| Total cholesterol (TC) (mmol/L) | 3.00-5.17 | 3.83±1.06 | 0.1760 | 3.85±0.80 | 0.0270^*^ | 3.79±0.87 | 0.0300^*^ |
| High-density lipoprotein (HDL) (mmol/L) | 1.03-1.55 | 1.05±0.36 | < 0.0001^***^ | 1.10±0.40 | < 0.0001^***^ | 1.04±0.35 | < 0.0001^***^ |
| Low-density lipoprotein (LDL) (mmol/L) | 0-3.10 | 2.43±1.20 | < 0.0001^***^ | 2.31±0.66 | < 0.0001^***^ | 2.30±0.72 | < 0.0001^***^ |
| Triglyceride (TG) (mmol/L） | 0.56-1.69 | 1.08±0.57 | 0.0670 | 0.97±0.41 | 0.9141 | 1.20±0.89 | 0.0200^*^ |

*P* values between TB cases with PYD, HFYD and DQY syndromes and the normal reference range were determined by one-sample *t*-test after taking the logarithm and comparison to the median.

**P*<0.05. ***P*<0.01. *** *P*<0.001
