# Supplementary material for: Microarray expression profile analysis of mRNAs and long non-coding RNAs in pulmonary tuberculosis with different traditional Chinese medicine syndromes
Source: BMC Complement Altern Med. 2016 Nov 17;16:472. doi: 10.1186/s12906-016-1436-y (PMC5114807; doi:10.1186/s12906-016-1436-y)
Supplement: Additional file 2: — Sample data for TB PYD, HFYD, and DQY patients and healthy controls. (DOCX 15 kb) [file 12906_2016_1436_MOESM2_ESM.docx]

**Additional File 2.** Sample data for TB PYD, HFYD, and DQY patients and healthy controls.

| **Specimen NO.** | **Age (years)** | **Gender** | **TCM syndrome** | **Sputum culture/smear** | **Sample use** |
| --- | --- | --- | --- | --- | --- |
| 1 | 50 | M | PYD | Positive | array 1 |
| 2 | 19 | F | PYD | Positive | array 1 |
| 3 | 58 | M | PYD | Positive | array 1 |
| 4 | 28 | F | PYD | Positive | array 1 |
| 5 | 61 | F | PYD | Positive | array 2 |
| 6 | 30 | M | PYD | Positive | array 2 |
| 7 | 25 | M | PYD | Positive | array 2 |
| 8 | 26 | F | PYD | Positive | array 3 |
| 9 | 23 | M | PYD | Positive | array 2 |
| 10 | 47 | M | PYD | Positive | array 3 |
| 11 | 18 | M | PYD | Positive | array 3 |
| 12 | 20 | M | HFYD | Positive | array 1 |
| 13 | 62 | M | HFYD | Positive | array 1 |
| 14 | 58 | M | HFYD | Positive | array 1 |
| 15 | 25 | F | HFYD | Positive | array 1 |
| 16 | 23 | F | HFYD | Positive | array 2 |
| 17 | 26 | F | HFYD | Positive | array 3 |
| 18 | 46 | F | HFYD | Positive | array 2 |
| 19 | 34 | M | HFYD | Positive | array 2 |
| 20 | 19 | M | HFYD | Positive | array 2 |
| 21 | 34 | M | HFYD | Positive | array 3 |
| 22 | 54 | M | HFYD | Positive | array 3 |
| 23 | 51 | M | DQY | Positive | array 1 |
| 24 | 51 | M | DQY | Positive | array 1 |
| 25 | 29 | F | DQY | Positive | array 1 |
| 26 | 23 | M | DQY | Positive | array 2 |
| 27 | 25 | F | DQY | Positive | array 1 |
| 28 | 64 | F | DQY | Positive | array 2 |
| 29 | 33 | F | DQY | Positive | array 2 |
| 30 | 38 | F | DQY | Positive | array 3 |
| 31 | 63 | F | DQY | Positive | array 3 |
| 32 | 51 | M | DQY | Positive | array 2 |
| 33 | 58 | M | DQY | Positive | array 3 |
| 34 | 46 | M | Control | Negative | array 1 |
| 35 | 32 | M | Control | Negative | array 1 |
| 36 | 53 | M | Control | Negative | array 1 |
| 37 | 34 | F | Control | Negative | array 1 |
| 38 | 34 | M | Control | Negative | array 2 |
| 39 | 44 | M | Control | Negative | array 2 |
| 41 | 42 | F | Control | Negative | array 2 |
| 42 | 30 | F | Control | Negative | array 2 |
| 40 | 44 | M | Control | Negative | array 3 |
| 43 | 28 | M | Control | Negative | array 3 |
| 44 | 28 | F | Control | Negative | array 3 |
